# Bridging Aging and Disability: A Replicable Framework for Inclusive Policy Design

**DOI:** 10.1093/geroni/igaf122.4079

**Published:** 2025-12-31

**Authors:** Sonya Durham, Annie Rhodes, Leland Waters

**Affiliations:** Virginia Commonwealth University, Richmond, Virginia, United States; Virginia Commonwealth University, Richmond, Virginia, United States; Virginia Commonwealth University, Richmond, Virginia, United States

## Abstract

States are now confronted with a critical policy gap, addressing the growing population of adults with intellectual and developmental disabilities (IDD). Aging services rarely account for the unique needs of these adults who face compounded health, economic, and social inequities. This project tackles this gap by adapting Missouri’s Master Plan on Aging (MPA) for Virginia with an emphasis on the IDD population. This approach will be used to reshape aging policy to be more inclusive, evidence-based, and replicable across diverse state contexts.

**Purpose:**

The Virginia Adaptation Report evaluates which components of Missouri’s MPA Needs Assessment can be transferred and which require modification, considering Virginia’s demographics, service systems, and policy landscape. This work includes disability within aging policy, applying innovative aging theories and model-building approaches.

**Methods:**

An environmental scan and gap analysis identified existing aging and disability resources and policies across Virginia. A concept map was developed to visualize service silos, theoretical omissions, and potential integration points. Innovation theory, equity-based models, and systems-thinking guided decisions on what to retain, adapt, or eliminate from the Missouri framework.

**Findings and Recommendations:**

The adaptation revealed structural and theoretical gaps in Virginia’s aging policy, particularly for aging individuals with IDD. Recommendations include implementing cross-sector data-sharing, integrating IDD-specific indicators into statewide aging surveys, expanding stakeholder engagement (including individuals with lived experience), and investing in inclusive training for service providers. By centering inclusive design and theory innovation, the resulting framework bridges traditional aging approaches with disability-inclusive strategies and provides a replicable, evidence-informed roadmap for other states.

